# Editorial: Biomarkers to predict, prevent and find the appropriate treatments of disorders in childhood

**DOI:** 10.3389/fped.2022.1093198

**Published:** 2022-11-23

**Authors:** Kristin Skogstrand, Nis Borbye-Lorenzen, Marie Bækvad-Hansen, Ulrik Lausten-Thomsen

**Affiliations:** ^1^ Danish Center for Neonatal Screening, Department for Congenital Disorders, Statens Serum Institut, Copenhagen, Denmark; ^2^ Neonatal Intensive Care Unit, Copenhagen University Hospital Rigshospitalet, Copenhagen, Denmark

**Keywords:** biomarkers, inflammatory markers, neonatal screening, genomics, pediatric, omics

**Editorial on the Research Topic** Biomarkers to predict, prevent and find the appropriate treatments of disorders in childhood

Biomarkers in its broadest sense refer to (bio)medical signs, i.e., an objective indication of a medical state, that can be accurately and reproducibly measured. Biomarkers play a huge role in clinical practice and research in adults, but for practical and ethical reasons the number of specific pediatric biomarkers has traditionally been fewer. However, neonatal and pediatric biomarkers absolutely can be used to predict, to prevent, or to diagnose disorders, and to find the right treatment, as well as to monitor treatment effects. Biomarkers can be prognostic, by predicting the recurrence of a disorder, or they can be predictive, by identifying which medicine is the best treatment for each patient as also described in The BEST (Biomarkers, EndpointS, and other Tools) guidelines (1). The current special issue presents a 2022 snapshot of pediatric biomarker research through 13 interesting and diverse papers:

Broadly speaking, markers for infection and/or inflammation is still very much a theme in biomarker research. This is exemplified in this Research Topic by papers exploring correlation between inflammatory markers and clinical conditions as variates. About half the included papers describe biomarkers reflecting the immune response and different conditions: Two papers, Brynge et al. and Fabricius et al., presents pediatric immunological markers' association to the mental disorders autism spectrum disorder and obsessive compulsive disorder, respectively. Biomarkers for psychiatric disorders is an underdeveloped field, and the complex and heterogenic topic of psychiatry would likely benefit immensely from the development of predictive, qualitative, and even diagnostic biomarkers, just as other areas of medicine have done over the last century or so. We have previously reported that biomarkers measured in samples taken a few days after birth associate with later diagnosis of autism spectrum disorder (2), but the biomarker differences were not significant enough to be used as a diagnostic or predictive tool. It is known that the causes of psychiatric disorders are multifactorial, but also broadly genetically dependent (3, 4), but the etiology for the different psychiatric disorders are largely unknown (5–7), and biomarkers can help with the understanding of the disorders' development. Thus, the field of psychiatry both regarding diagnostics and treatments is solely based on symptoms, which can lead to both under- and over-diagnosis, as well as ineffective treatments due to lack of knowledge in personal medicine (8–10). We believe and hope that in the near future, biomarkers will become an important part of psychiatry (2–4).

Inflammatory markers were also analyzed in Faust et al.'s paper, describing a correlation between neonatal inflammation and bronchopulmonary dysplasia, and Ouyang et al. used inflammatory markers as a prognostic tool for pediatric osteosarcoma.

Lamot et al. describes biomarkers as a tool for separating viral from bacterial infections, and Feketea et al. have found a correlation between vitamin D and mean platelet volume in children with viral respiratory infections.

Rossi et al. discusses an important subject for several neonatal screening disorders; how to separate diagnosis with symptoms to asymptomatic cases. As the laboratory technologies get more sensitive, and we are able to analyze about everything in a few drops of blood, the number of disorders in neonatal screening panels all over the world are increasing (11, 12). When is a screen positive sample actually synonymous with a disorder in the child is thus a question more important than ever before. For many disorders, biochemical analyses may be followed up by genotyping to reduce the number of false positive samples (13). This is though not always possible, as the consequence of different genetic variations and combinations sometimes are not known. Thus, the possibility of screening newborns for multiple disorders should be carefully balanced through ethical considerations such as the risk of making otherwise asymptomatic children sick due to a false positive screening result.

Genetics and outcome are presented in one paper by Liu et al., looking at risk for hypospadias with different gene polymorphisms. Xiang et al. have looked at environmental factors by analyzing urine phthalate associations to adolescents' liver function. Mingwen et al. presents the only paper in this issue using biomarkers not measured in body fluids, being parent-reported measures of sleep patterns, to find a model to classify sleep disorders.

Our research topic contains one review paper by Nguyen et al., where the authors have reviewed metabolomics results and lung exacerbations in cystic fibrosis children, and one paper by Tao et al., exploring predictors for syncopal recurrence in children treated with metoprolol. Miller et al. presents another important biomarker topic; biomarkers to predict prognosis after head injury.

As encouraging as these papers are, generally in the area of biomarker research, there is often far between the manuscripts concluding with clinically relevant biomarkers. Several biomarkers may be statistically significant, but cannot be used as either screening or diagnostic markers. The perfect diagnostic biomarker, that is, a biomarker with 100% sensitivity and specificity, does not exist, but biomarkers with very low specificity are of poor value for diagnostic purposes. All statistically significant biomarkers may though help in the understanding of the disorders' etiology. A growing trend is quantity over quality, that is, the more markers the better, employing modern high-throughput laboratory techniques called omics, e.g., genomics, proteomics, transcriptomics, metabolomics, and microbiomics methods. The impressive progress in the field has enabled the fast discovery of candidate biomarkers and consequently large numbers of preclinical reports have been published. The relative complexity of these technologies put extra stress on requirement for well-designed biomarker discovery processes to develop clinically relevant biomarkers. The challenge then is to sort out the useful markers, and to make a mathematic formula that is more than just statistically significant, but also actually clinically useful.

When a biomarker has the potential to become a predictive or diagnostic biomarker, biobank resources such as those found in Denmark and other Scandinavian countries with a wealth of accessible health registers become highly relevant to prove the biomarkers potential (14, 15).

Study design is very important during exploration for biomarkers. Controls should be selected carefully, not only regarding gender, age and BMI, but also regarding treatments, populations etc., and the statistical calculations should be made by people who actually know what they are doing. The more biomarkers available, the more complex the calculations get. Using the wrong statistical methods, almost all studies will find statistically significant biomarkers.

Particularly genomics has clinical appeal: Why use biomarkers in the form of proteins or smaller molecules, when the whole human genome is available? Is it possible to solely use genetic variation either as biomarker for disorders or for personal medicine in the future? Can a few drops of blood and a whole genome sequencing test be enough in the future to both set the diagnosis and to choose the best medicine for the patient? For a few disorders it might be sufficient, but in the majority of cases probably not, as most disorders cannot be explained by genetic variation alone. The lack of effect of medicines can be caused by other molecules in the blood, e.g., environmental factors, or by a combination of both external factors and genetics. In addition, there is the layer of posttranslational modification that is highly tissue-dependent and not directly predictable through a genomic analysis alone (16). The combination of physical symptoms and blood biomarkers, both protein-based and genetic, are probably for most disorders the best diagnostic combination to avoid both under- and over-diagnosis. Some people claim that all humans can get at least one diagnosis if we get examined thorough enough (17), and this is obviously not what we want as a society. Thus, we are not done yet in the research for good, useful biomarkers for disorders.
